# Multi-infusion with integrated multiple pressure sensing allows earlier detection of line occlusions

**DOI:** 10.1186/s12911-021-01668-7

**Published:** 2021-10-28

**Authors:** Frank Doesburg, Roy Oelen, Maurits H. Renes, Pedro M. Lourenço, Daan J. Touw, Maarten W. Nijsten

**Affiliations:** 1 Department of Critical Care, University of Groningen, University Medical Center Groningen, Huispostcode TA29. Hanzeplein 1, 9713 GZ Groningen, The Netherlands; 2 Hanze Institute of Engineering, Industrieweg 1, 9402 NP Assen, The Netherlands; 3 Department of Clinical Pharmacy and Pharmacology, University of Groningen, University Medical Center Groningen, Hanzeplein 1, 9713 GZ Groningen, The Netherlands; 4 Groningen Research Institute of Pharmacy, Department of Pharmaceutical Analysis, University of Groningen, Hanzeplein 1, 9713 GZ Groningen, The Netherlands

**Keywords:** Infusion, Intravenous, Algorithms, Infusion pumps, Multi-infusion, Occlusion, Co-occlusion

## Abstract

**Background:**

Occlusions of intravenous (IV) tubing can prevent vital and time-critical medication or solutions from being delivered into the bloodstream of patients receiving IV therapy. At low flow rates (≤ 1 ml/h) the alarm delay (time to an alert to the user) can be up to 2 h using conventional pressure threshold algorithms. In order to reduce alarm delays we developed and evaluated the performance of two new real-time occlusion detection algorithms and one co-occlusion detector that determines the correlation in trends in pressure changes for multiple pumps.

**Methods:**

Bench-tested experimental runs were recorded in triplicate at rates of 1, 2, 4, 8, 16, and 32 ml/h. Each run consisted of 10 min of non-occluded infusion followed by a period of occluded infusion of 10 min or until a conventional occlusion alarm at 400 mmHg occurred. The first algorithm based on binary logistic regression attempts to detect occlusions based on the pump’s administration rate Q(t) and pressure sensor readings P(t). The second algorithm continuously monitored whether the actual variation in the pressure exceeded a threshold of 2 standard deviations (SD) above the baseline pressure. When a pump detected an occlusion using the SD algorithm, a third algorithm correlated the pressures of multiple pumps to detect the presence of a shared occlusion. The algorithms were evaluated using 6 bench-tested baseline single-pump occlusion scenarios, 9 single-pump validation scenarios and 7 multi-pump co-occlusion scenarios (i.e. with flow rates of 1 + 1, 1 + 2, 1 + 4, 1 + 8, 1 + 16, and 1 + 32 ml/h respectively). Alarm delay was the primary performance measure.

**Results:**

In the baseline single-pump occlusion scenarios, the overall mean ± SD alarm delay of the regression and SD algorithms were 1.8 ± 0.8 min and 0.4 ± 0.2 min, respectively. Compared to the delay of the conventional alarm this corresponds to a mean time reduction of 76% (*P* = 0.003) and 95% (*P* = 0.001), respectively. In the validation scenarios the overall mean ± SD alarm delay of the regression and SD algorithms were respectively 1.8 ± 1.6 min and 0.3 ± 0.2 min, corresponding to a mean time reduction of 77% and 95%. In the multi-pump scenarios a correlation > 0.8 between multiple pump pressures after initial occlusion detection by the SD algorithm had a mean ± SD alarm delay of 0.4 ± 0.2 min. In 2 out of the 9 validation scenarios an occlusion was not detected by the regression algorithm before a conventional occlusion alarm occurred. Otherwise no occlusions were missed.

**Conclusions:**

In single pumps, both the regression and SD algorithm considerably reduced alarm delay compared to conventional pressure limit-based detection. The SD algorithm appeared to be more robust than the regression algorithm. For multiple pumps the correlation algorithm reliably detected co-occlusions. The latter may be used to localize the segment of tubing in which the occlusion occurs.

*Trial registration* Not applicable.

**Supplementary Information:**

The online version contains supplementary material available at 10.1186/s12911-021-01668-7.

## Background

Occlusions of intravenous (IV) tubing can prevent vital and time-critical medication from being delivered into the bloodstream of patients. Conventional algorithms to detect occlusions sound an alarm when the pressure measured by the pump exceeds a certain threshold. A low pressure threshold will detect an occlusion sooner at the expense of an increase in the likelihood of false alarms, which is a known contributor to alarm fatigue [1]. Conversely, a high threshold will cause occlusions to be detected later, albeit with a decreased rate of false alarms. Using conventional pressure threshold algorithms it can take nearly up to 2 h before an alarm is activated when administration rates are low (≤ 1 ml/h) [2]. The use of higher rates (e.g. > 10 ml/h) and low-compliance IV tubing may decrease the alarm delay, but many critical drugs are restricted to lower administration rates [3].

**Fig. 1:**
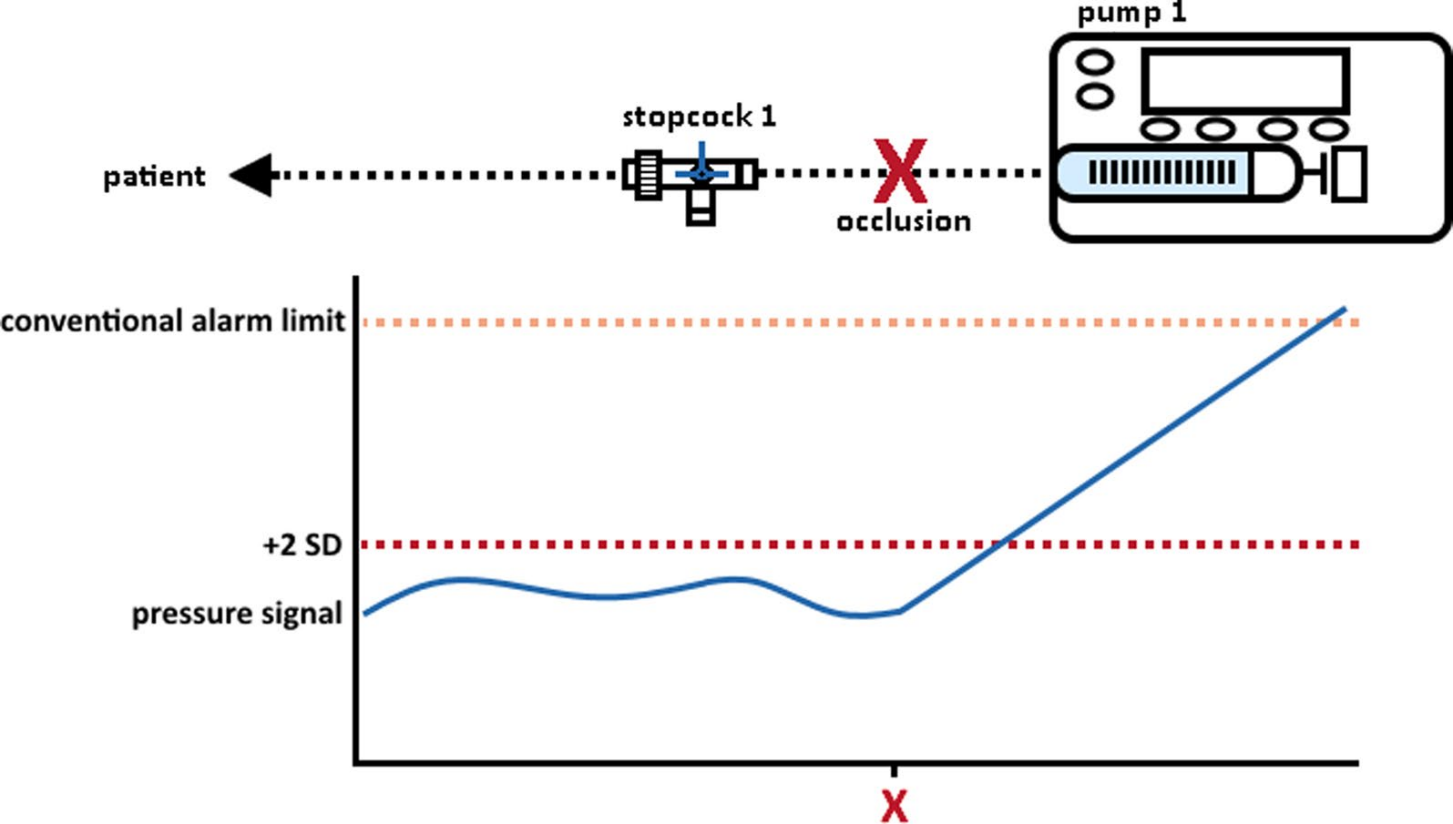
Single-pump occlusion detection using the standard deviation (SD). During stable infusion pressure values are assumed to deviate less than 2 times the SD from the mean pressure value. When the pressure exceeds the threshold set at twice the SD, an occlusion is likely

Common statistical methods may be used to reduce alarm delays compared to conventional pressure threshold algorithms. One approach may use a binary logistic regression model, which describes the relationship of a binary (occlusion vs. non-occlusion) outcome with one or more predictors [4]. Based on the pump’s administration rate Q(t) (ml/h) and its pressure P(t) (mmHg) such a model may be able to detect the occurrence of an occlusion. Another method could use the standard deviation (SD) of the a set of recent values of P(t) to define a real-time threshold for anomalies that occur during an occlusion (Fig. 1). During stable infusion and assuming a normal distribution, 95% of pressure values will deviate less than 2 times the standard deviation (SD) from the mean pressure value [5]. A threshold to detect a pressure anomaly could be set at twice that SD. This approach requires the measurement of P(t) and SD during stable infusion at different administration rates.

With increasing complexity of patients, multi-infusion systems are increasingly employed. Such systems are increasingly integrated with respect to monitoring and control of multiple pumps from a centralized single controller [6]. When the number of infusion pumps is larger than the number of available intravenous (IV) access points several pumps have to be connected to the same IV access point [7]. In case of an obstruction in the pathway between such pumps and the patient, one or more pumps may be affected by the obstruction, depending on where the physical obstruction is present.

**Fig. 2:**
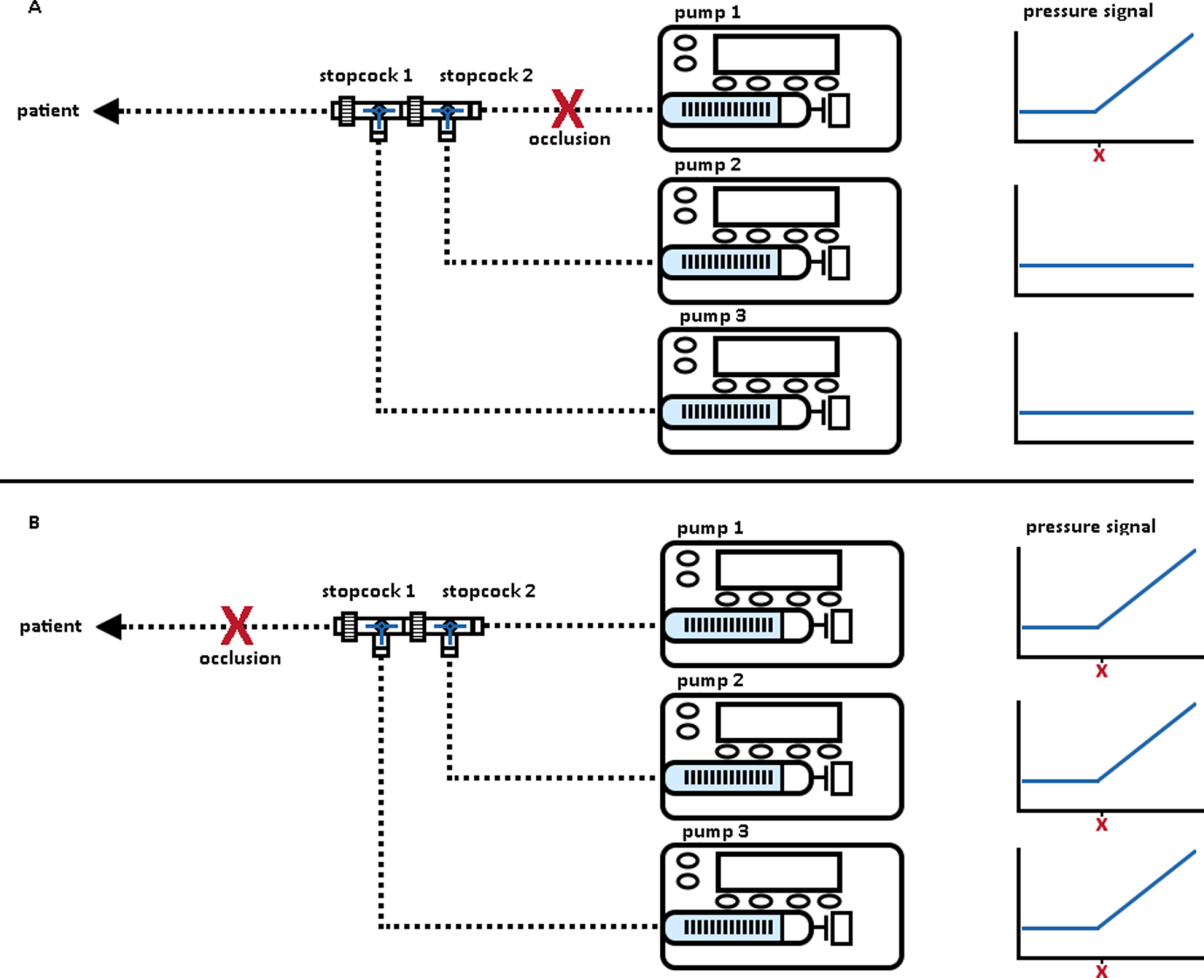
Multi-pump and single-pump occlusions in a multi-infusion set-up. During an occlusion in the shared pathway between the pumps the pressures measured by each pump will rise coincidently (Panel **A**). During a single-pump occlusion the pressure in only one pump is expected to rise, while the pressure of the other pumps remains stable (Panel **B**)

In multi-infusion settings combining the pressure signals P1(t), P2(t), etc. from several pumps might allow both earlier and more specific detection of obstructions in a common line to the patient. In case the pressures in two or more pumps that deliver to the same IV access show a coincident rise, then an obstruction in the final common pathway is likely (Fig. 2B). In case the pressure of only one pump increases, this suggests an obstruction in the infusion line between that specific pump and the common infusion line (Fig. 2A). In case the pressures in two or more pumps that deliver to the same IV access show a coincident rise, then an obstruction in the final common pathway is likely (Fig. 2B).

In this study we aimed to reduce the alarm delay in single and multi-pump occlusion scenarios while maintaining a high level of accuracy. We therefore developed and tested two occlusion detection algorithms for single pumps using common statistical methods. Secondly, we developed and tested an algorithm designed to detect co-occlusions in IV tubing by correlating the pressure signals in multiple pumps, thereby providing a framework for occlusion localization. To our knowledge no studies have previously assessed the feasibility of a multi-infusion system to detect and pinpoint occlusions.

## Methods

### Materials

Three Alaris Asena GH Syringe pumps (Carefusion, United Kingdom) with firmware v2.3.6 were connected to an Alaris DS docking station. A generic laptop running Windows 10 (Microsoft Corporation, United States of America) was used to run custom pressure logging software written in Java 1.7 (Oracle Corporation, United States of America). Algorithm evaluation software was also written in the Java environment. Communication between the computer and the pumps followed the pumps’ RS232 communication protocol [8]. A StarTech ICUSB2324X USB to serial adapter (StarTech, United Kingdom) and 3 generic RS232 cables were used for RS232 connectivity.

Tubing for the baseline and co-occlusion measurements consisted of two Steritex 3 W three-way stopcocks (Codan, Denmark), three Vygon VGreen IV (2 m length, 2 ml volume, 1 mm internal diameter) tubes (Vygon, France), and an Arrow-Howes MC-12703 triple-lumen central venous catheter (Teleflex Inc., United Kingdom). Three BD Plastipak 50 ml syringes (Becton–Dickinson, United States of America) were used. A generic plastic waste container was used as the end point of the catheter.

Tubing for the validation measurements consisted of three BD Plastipak 20 ml syringes, three Fresenius Injectomat 50 ml syringes (Fresenius Kabi, France), three Codan 71.4021 flexible tubes (1.5 m length, 10.6 ml volume, 3 mm internal diameter; Codan, Denmark), and three MPH Medical Devices 12040150E (1.5 m length, 1.2 ml volume, 1 mm internal diameter; MPH Medical Devices, Czech Republic). The same brand and type of stopcocks, catheter and waste container used in the baseline measurements were also used in the validation measurements.

### Experimental setup

Three infusion pumps were attached to a docking station in a vertically stacked fashion. The docking station was attached to a wall so that the middle pump was at approximately the same height as the tip of the triple-lumen catheter. The three syringes were filled with 50 ml of tap water and connected to an IV tube. Subsequently, two stopcocks were connected to the tubes. One stopcock connected to the distal (16 Ga., 0.39 ml priming volume) lumen of the catheter. The tip of the catheter was submerged in approximately 7 cm of tap water, corresponding to a counter pressure of 5.2 mmHg simulating a normal central venous pressure (2–8 mmHg) [9, 10]. The syringes were placed in the pumps, followed by priming of the tubing using the pumps’ built-in priming functionality until there was no more air present in the tubing.

### Gathering of experimental data for the development and evaluation of the algorithms

#### Baseline single-pump scenarios

In order to develop a reference set of baseline pressure characteristics experimental runs were recorded using three separate pumps simultaneously. Runs were recorded in triplicate at administration rates of 1, 2, 4, 8, 16, and 32 ml/h and consisted of two phases. The first phase consisted of ten minutes of non-occluded infusion. Subsequently an occlusion phase was started by closing both stopcocks and clicking a designated button in the logging software that created a timestamped occlusion event entry. Every 2 s the software generated a log entry for each pump which contained a pump-identifier (numbers 0, 1, and 2), a timestamp, the administration rate (ml/h), an event code (no-event or occlusion event), and the pressure as measured by the pumps’ internal sensors (mmHg). The experiment ended after ten minutes of occlusion or when an occlusion alarm was generated by the pump.

After recording the experimental runs the pressure log files were examined by testing for a normal distribution, and the baseline characteristics (pressure mean and standard deviation for each rate) were calculated for the non-occluded phase. In case the pump was not immediately pressurized at the start of a run (i.e. the pressure was still rising to a stable pressure), those ‘start-up’ values were omitted from the calculation of the baseline characteristics.

Linear regression was used to determine the pressure increase per second during occlusions at different administration rates during the occlusion phase. The resulting regression lines were used to calculate the alarm delay (time from the start of the occlusion to the alarm) for a range of alarm thresholds found in literature (300–800 mmHg) assuming a ‘worst case’ baseline pressure of 150 mmHg [3, 11–13].

#### Validation scenarios

To assess the robustness of the single-pump algorithms nine additional runs were recorded in triplicate using a method that was similar to that of the baseline measurements, but with different syringes, tubing and rates. In three runs a smaller (20 ml) syringe was used together with 150 cm thin, rigid tubes at rates of 1, 5, and 25 ml/h. In another three runs a 50 ml syringe from a different manufacturer was used together with 150 cm thin, rigid tubes at rates of 1, 5, and 25 ml/h. To assess the algorithms’ robustness in a volumetric (large volume) infusion scenario, three runs were recorded with a 50 ml syringe and thick (3 mm) flexible tubing at rates of 25, 50 and 75 ml/h.

#### Experimental data for the evaluation of co-occlusions

**Fig. 3:**
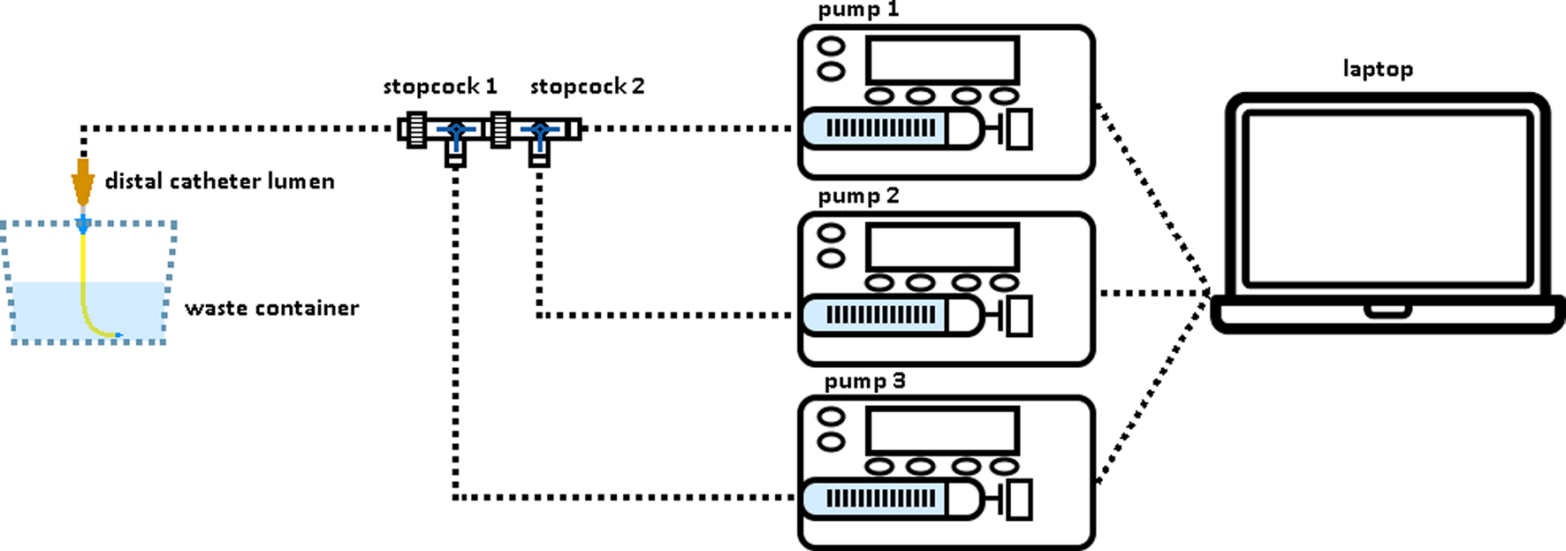
Experimental setup

A series of experimental runs was performed in triplicate where two pumps were co-occluding on the same lumen. The same experimental procedure and setup used to collect the baseline single-pump measurements was followed, with a few differences: The administration rate of one pump was kept constant at 1 ml/h, and the rate of the second pump was varied in each run, resulting in rates of 1 + 1, 1 + 2, 1 + 4, 1 + 8, 1 + 16, and 1 + 32 ml/h. Pump 3 (Fig. 3) was not used and during the non-occlusion phase stopcock 1 was configured so that water could not flow from or towards pump 3. Finally, in order to create a shared occlusion only stopcock 1 (Fig. 3) was closed instead of all stopcocks.

### Real-time occlusion detection algorithms

#### Regression algorithm

Two single-pump detection algorithms were created and evaluated. The first detection algorithm involves a binary logistic regression model. The (rounded) output of such a model is either 0 or 1, which makes it suitable for a binary classification (non-occlusion vs. occlusion). The regression model was trained using the data collected using baseline single-pump occlusion scenarios. Cases were labeled as corresponding to a non-occlusion, or an occlusion (the dependent variable). Independent variables were administration rate Q(t) (ml/h) and pressure P(t) (mmHg). This resulted in the following regression model:

$$Output=\frac{1}{1+ {e}^{-(-1.345 -0.177*Q(t) + 0.040*P(t))}}$$

where a rounded output of 1 corresponds to an occlusion, and 0 to a non-occlusion.

**Fig. 4:**
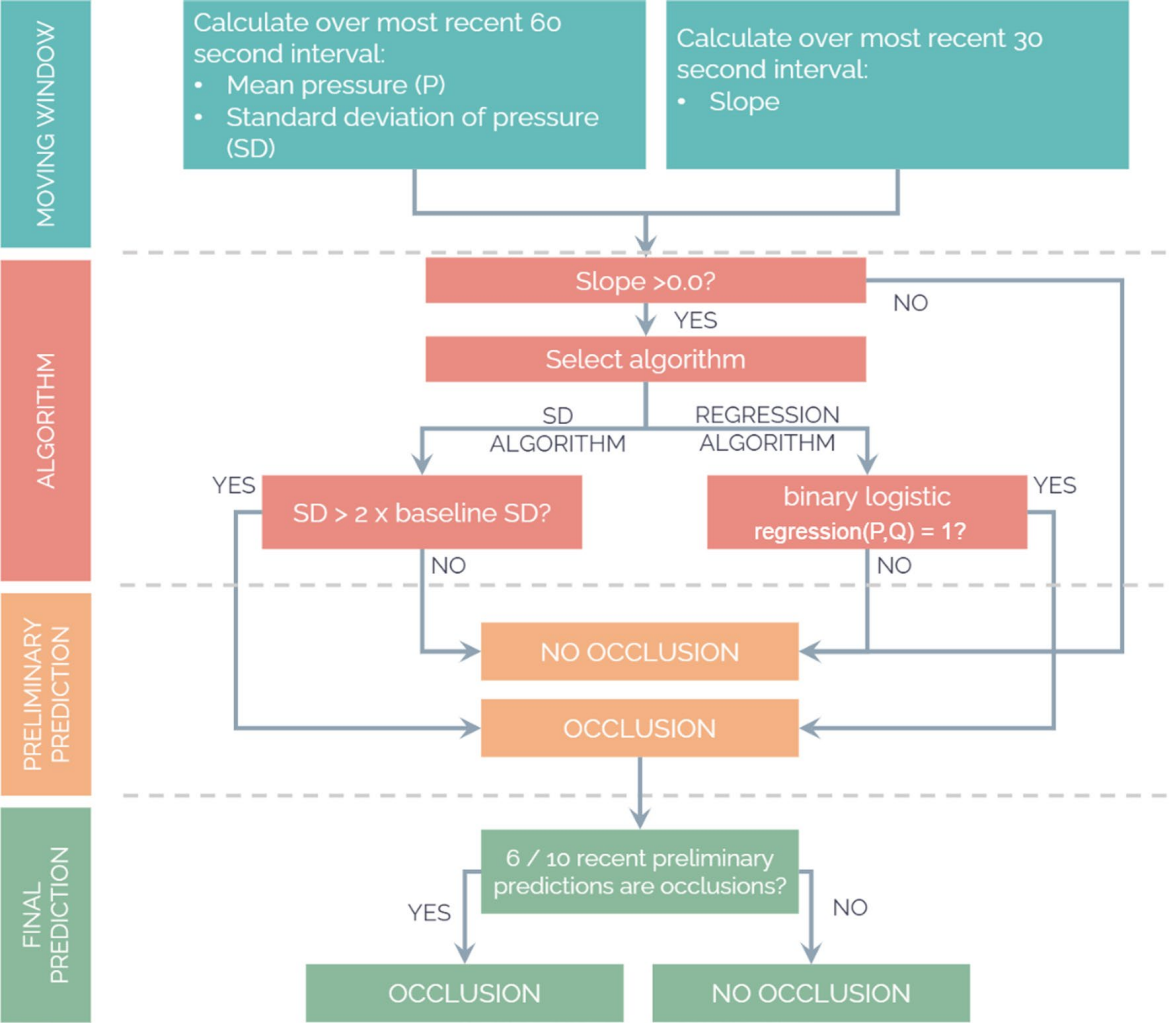
Flow chart of the two single-pump occlusion detection algorithms

The regression algorithm first checks for a positive slope in pressure over the most recent 30 s interval, and then enters the rate and mean pressure over a 60 s interval into the formula above (Fig. 4). The size of the window was determined by testing window sizes between 2 and 60 s and selecting the size that had the best accuracy (%; Additional file 1). An output of 1 produces a positive preliminary occlusion classification corresponding to a single time point. In order to reduce sensitivity to outliers and noise, at least 6 of the 10 most recent preliminary classifications had to be positive to produce a final positive occlusion classification (i.e. an actual alarm).

#### Standard deviation (SD) algorithm

The SD algorithm assumes that pressures measured by the pump during non-occluded infusion are normally distributed around the mean pressure. In that case 95% of these values are within 2 SDs from the mean [5]. The SD algorithm calculates the SD over a moving window of 30 measurements (60 s) and compares it to the reference set with baseline SD’s (Fig. 4). If the pressure’s slope is positive and the window’s SD is larger than twice the baseline SD, this produces a positive preliminary occlusion classification. Similar to the regression algorithm, 6 out of the 10 most recent preliminary classifications had to be positive to produce a final positive occlusion classification.

#### Correlation algorithm

The purpose of the correlation algorithm was to detect co-occlusions using the correlation between pressure measurements from multiple pumps. The correlation algorithm was only used when the SD algorithm made a final positive occlusion classification. When that was the case, Pearson correlations were calculated over an interval of 30 measurements (60 s), thus correlating 30 measurements from one pump with 30 measurements from another pump. If the correlation between the pressures of two pumps was > 0.8, the final classification of the correlation algorithm was that both pumps were co-occluding.

### Evaluation of algorithms

All three algorithms were programmed into custom algorithm evaluation software. The software was able to process pump log files, run each algorithm and export a report that detailed the performance of each algorithm in terms of alarm delay (min) and accuracy (correct/incorrect classifications). The software also exported a.csv file for each pump with the preliminary and final occlusion classifications from the algorithm as well as correlations with other pump pressures at each time point.

The baseline single-pump measurement log files were used to evaluate the performance of the regression and SD algorithms at administration rates of 1, 2, 4, 8, 16, and 32 ml/h. To evaluate the robustness of both single-pump algorithms a set of validation measurements was created with varying syringes (size and brand), IV tubing and rates.

The correlation algorithm was evaluated using the log files of the multi-pump scenarios. At the start of a measurement the pressure was not always immediately stable (e.g. P(t) was still increasing to a stable level), and in such case the unstable interval was omitted from analysis. The primary performance measure was alarm delay (min).

### Statistics

IBM SPSS Statistics V23.0 for Windows (IBM Corporation, Armonk, NY) was used for the statistical analysis. When normally distributed the mean ± standard deviation (SD) are presented, otherwise the median and interquartile range (IQR) are shown.

By default, statistical significance was concluded at a two-sided p-value < 0.05. In the single pump scenarios overall statistically significant differences in alarm delay between the regression and SD algorithms were determined using the Student’s t-test. Comparisons of alarm delays of the regression and SD algorithms vs. conventional pressure threshold levels (300–800 mmHg) were evaluated using paired t-tests.

In the multi-pump scenarios, the alarm delay of the three algorithms was compared using a one way analysis of variance (ANOVA) with post hoc Bonferroni tests at a corrected significance level of 0.05/3 = 0.017.

## Results

### Baseline single-pump scenarios

**Fig. 5:**
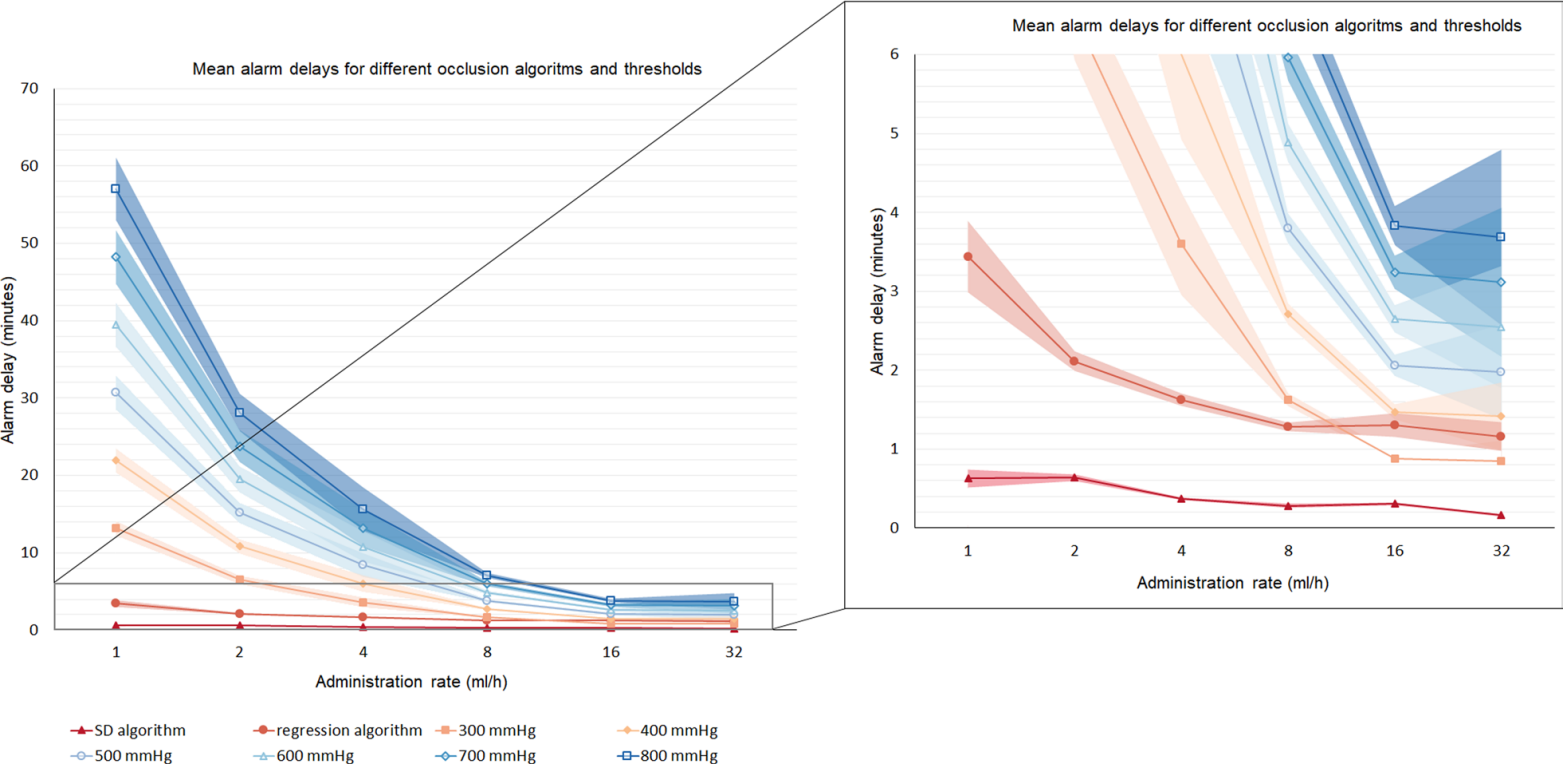
Alarm delays for the baseline single-pump scenarios. Mean ± SD alarm delays for conventional alarm thresholds (300–800 mmHg) and the SD and regression algorithms in physical pump occlusion scenarios at different administration rates. Data points for the conventional alarm thresholds were extrapolated from the baseline data. A detailed view is shown on the right-hand side

Experimental alarm delays for the regression and SD algorithms, and for the calculated conventional pressure limits between 300 and 800 mmHg are shown in Fig. 5. Numerical values for all conventional pressure limits corresponding to Fig. 5 are listed in Additional file 2. Pairwise comparisons between alarm delays of the regression and SD algorithms with our local alarm limit of 400 mmHg at different administration rates are listed in Table 1.

**Table 1:** Alarm delays in minutes for the regression and SD algorithms compared to calculated conventional alarm delays with an alarm threshold set at 400 mmHg in baseline single-pump scenarios

Rate (ml/h)	Detection algorithm	Alarm delay mean ± SD (minutes)	Conventional alarm delay mean ± SD (minutes)^1^	*P*-value^2^
1	SD	0.6 ± 0.1	21.9 ± 1.9	< 0.01
	Regression	3.4 ± 0.6		< 0.01
2	SD	0.6 ± 0.1	10.8 ± 1.1	< 0.01
	Regression	2.1 ± 0.2		< 0.01
4	SD	0.4 ± 0.0	6.0 ± 1.1	< 0.01
	Regression	1.6 ± 0.1		0.03
8	SD	0.3 ± 0.0	2.7 ± 0.2	< 0.01
	Regression	1.3 ± 0.1		< 0.01
16	SD	0.3 ± 0.0	1.5 ± 0.1	< 0.01
	Regression	1.3 ± 0.2		0.88
32	SD	0.2 ± 0.0	1.4 ± 0.5	0.05
	Regression	1.2 ± 0.2		0.54
Overall	SD	0.4 ± 0.2	7.4 ± 7.5	< 0.01
	Regression	1.8 ± 0.8		< 0.01

^1^Calculated alarm delay for our local threshold setting of 400 mmHg

^2^Paired Student t-test

All scenarios were performed in triplicate

The overall mean ± SD alarm delay of the regression and SD algorithms were 1.8 ± 0.8 min and 0.4 ± 0.2 min, respectively. Compared to the delay of the conventional alarm of 7.4 ± 7.5 min this corresponds to a mean time reduction of 76% (P = 0.003) and 95% (P = 0.001), respectively.

There were no false negative classifications (undetected occlusions) by our algorithm. Likewise, there were no false positive alarms in the baseline single-pump scenarios.

### Validation scenarios

In the validation scenarios the overall mean ± SD alarm delay for the regression algorithm was 1.8 ± 1.6 min compared to a conventional alarm delay of 7.7 ± 13.0 (*P* < *0.05*), which corresponds to a reduction in alarm delay of 77%. The overall mean ± SD alarm delay for the SD algorithm was 0.3 ± 0.2 min compared to a conventional alarm delay of 6.2 ± 12 (*P* < *0.05*), which corresponds to a reduction in alarm delay of 95%.

Pairwise comparisons between alarm delays of the regression and SD algorithms with our local alarm limit of 400 mmHg at different administration rates are listed in Table 2. Using the regression algorithm false negatives (i.e. the algorithm was not able to detect an occlusion before a conventional alarm) occurred in 2 scenarios (using a 20 ml syringe at 25 ml/h and using a thick IV tube at 75 ml/h). In 2 runs the SD algorithm classified a (temporarily) increased pressure as an occlusion, which overlapped with the actual onset of an occlusion. These two runs were omitted from Table 2.

**Table 2:** Validation set alarm delays in minutes for the regression and SD algorithms compared to calculated conventional alarm delays with an alarm threshold set at 400 mmHg

Syringe (product name, internal volume)	IV tube (product name, internal diameter, length)	N	Rate (ml/h)	Detection algorithm	Alarm delay mean ± SD (minutes)	Conventional alarm delay mean ± SD (minutes)^1^	*P*-value^2^
BD Plastipak 20 ml	MPH Medical Devices 12040150E, Ø 1.0 mm, 1500 mm	2^3^	1	SD	0.3 ± 0.2	5.9 ± 1.2	0.08
		3		Regression	1.1 ± 0.3	6.3 ± 1.1	< 0.05
		2^3^	5	SD	0.3 ± 0.1	1.5 ± 0.5	0.20
		3		Regression	0.9 ± 0.1	1.4 ± 0.4	0.18
		3	25	SD	0.2 ± 0.0	0.3 ± 0.1	0.06
				Regression	N.A.^4^		N.A
Fresenius Injectomat 50 ml	MPH Medical Devices 12040150E, Ø 1.0 mm, 1500 mm	3	1	SD	0.7 ± 0.2	37.4 ± 9.9	< 0.05
		3		Regression	5.4 ± 1.9	37.4 ± 9.9	< 0.05
		3	5	SD	0.4 ± 0.0	5.0 ± 0.7	< 0.01
		3		Regression	1.5 ± 0.1	5.0 ± 0.7	< 0.01
		3	25	SD	0.3 ± 0.0	1.1 ± 0.2	< 0.05
		3		Regression	1.2 ± 0.2	1.1 ± 0.2	< 0.05
Fresenius Injectomat 50 ml	Codan 71.4021, Ø 3.0 mm, 1500 mm	3	25	SD	0.4 ± 0.0	1.9 ± 0.1	< 0.01
		3		Regression	1.4 ± 0.1	1.9 ± 0.1	< 0.05
		3	50	SD	0.3 ± 0.0	0.8 ± 0.0	< 0.001
		3		Regression	1.4 ± 0.1	0.8 ± 0.0	< 0.01
		3	75	SD	0.2 ± 0.0	0.5 ± 0.0	< 0.001
				Regression	N.A.^4^		N.A

^1^Calculated alarm delay for our local threshold setting of 400 mmHg

^2^Paired Student t-test

^3^A case was excluded due to the occurrence of a false positive that overlapped with the onset of the occlusion

^4^The regression algorithm was not able detect an occlusion in this case (i.e. a false negative classification occurred)

In order to test the robustness of the algorithms, syringes and IV tubes were used that were different from those used to develop the algorithms

### Multi-pump scenarios

In the multi-pump scenarios, the overall mean ± SD alarm delay of the SD algorithm (0.4 ± 0.2 min) and the correlation algorithm (0.4 ± 0.2 min) was lower than that of the regression algorithm (2.1 ± 0.9 min), *P* < *0.001* in both cases. The difference in alarm delay between the SD and the correlation algorithms was not significant. False negative classifications did not occur. One false positive alarm occurred in a scenario with a combined rate of 17 ml/h. In this particular case the SD alarm was triggered for a period of 14 s during a brief fluctuation in pressure, after which the algorithm self-corrected.

**Fig. 6:**
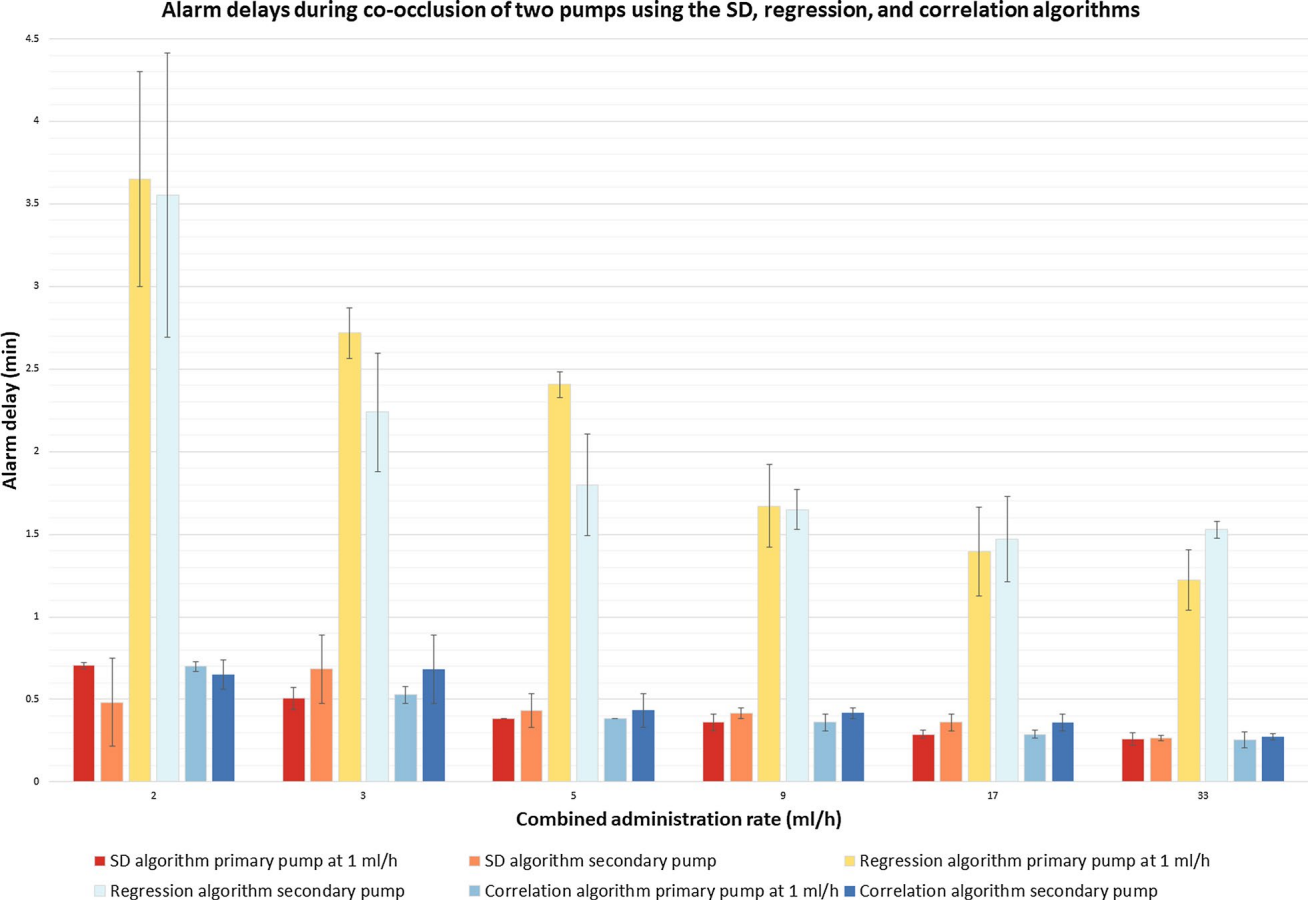
Alarm delays for two pumps during a co-occlusion. Mean ± SD alarm delays during co-occlusions using the SD, regression and correlation algorithms. The primary pump was running at a rate of 1 ml/h in every scenario, while the rate of the secondary pump was increased, resulting in increasing combined rates

Figure 6 shows the alarm delays for two pumps using the SD, correlation and regression algorithms.

## Discussion

In this study we aimed to develop and test the performance of two single-pump occlusion detection algorithms and one multi-pump occlusion detection algorithm. In the single-pump scenarios we found that both the regression and SD algorithms were able to detect occlusions much faster than conventional pressure threshold algorithms. The SD algorithm was both faster and more accurate than the regression algorithm. In the multi-pump scenarios, the SD and correlation algorithms showed a similar performance, and both were faster and more accurate than the regression algorithm.

In de baseline scenarios the mean time reduction compared to our local alarm threshold of 400 mmHg was 76% and 95% respectively using the regression and the SD algorithms. The reduction in alarm delay was significant at administration rates ≤ 8 ml/h. This is an important result as conventional occlusion detection generally has a poor performance at low rates [2]. The mean time reduction was similar in the validation scenarios (i.e. 77% and 95% using the regression and SD algorithms, respectively.

The robustness of the regression and SD algorithms was tested in the validation scenarios. In three scenarios a smaller syringe was used that had a lower compliance compared to the more common 50 ml syringes. Our data shows that pressures increased more rapidly using the smaller syringe, and ‘spontaneous’ pressure fluctuations appeared to be more frequent as well. This may explain why false positives occurred in two validation scenarios using the SD algorithm. Using the SD algorithm false negative classifications did not occur. If there was an occlusion, it was always detected by the SD algorithm before our local conventional alarm limit of 400 mmHg was reached. In 2 validation scenarios false negative classifications occurred using the regression algorithm. In both scenarios the administration rate was relatively high (i.e. 25 and 75 ml/h) and either a low compliance syringe or a high compliance IV tube was used. From these observations we conclude that the SD algorithm is more robust when different disposables are used compared to the regression algorithm. Nevertheless there is still room to improve the SD algorithm as it appears be more sensitive to fluctuations in pressure than the regression algorithm.

To our knowledge there are no existing studies that investigated the feasibility of occlusion localization using the pressure measurements from multiple pumps. Our correlation algorithm was able to detect a co-occlusion with a high degree of accuracy at different combinations of administration rates. When two pumps show a coincident rise in pressure, it is likely that both pumps are affected by an occlusion located in a segment of the IV tubing that is shared between them. Such information may help nurses to pinpoint and resolve occlusions faster.

The fact that our current design obtains pressures every 2 s may seem an overly high sampling frequency. But in particular in multi-pump set-ups this can lead to far more rapid detection of occlusions.

During the co-occlusion of both pumps the tubing acts like a closed system with pumps at both sides. In a static closed system with fluid, Pascal’s law holds. It states that pressure exerted on the fluid is transmitted almost instantaneously in all directions [14]. Therefore, using a high pressure sampling frequency makes sense and allows for rapid detection of a correlation between pressure changes detected by multiple pumps.

In the multi-pump experiments the primary pump was running at the same rate (1 ml/h) in each scenario. As the combined rate increased, the alarm delay of the primary pump decreased (Fig. 6). The primary pump at 1 ml/h will detect a much larger pressure increase caused by the second pump compared to a single-pump occlusion. The detection delay of a pump running at a low rate becomes shorter when it shares tubing with another pump running at a higher rate.

In our experiments the counter pressure consisted of the tubing’s compliance and an artificial central venous pressure of approximately 5.2 mmHg [10]. Depending on the use of additional disposables such as filters, anti-siphon and anti-reflux valves, counter pressure may be as high as 50 mmHg in neonates and 150 mmHg in adults [15]. As our algorithms only take the deviation from the mean pressure into account (and not the absolute value of the mean itself) during occlusion detection, the alarm delay will still be relatively short, regardless of the mean baseline pressure.

The time until a stable pressure is reached will be proportional to the alarm delay as they both are influenced by the pumping mechanism, syringe and tubing compliance, and the administration rate^11^. The data in Table 2 provide an indication how compliance and rate may affect the stabilization time. For example, the conventional alarm delay was up to six times larger when a 50 ml syringe was used instead of a lower compliance 20 ml syringe. In such a case a longer stabilization time is to be expected as well. In our experiments we reduced the stabilization time by priming the tubing prior to each run using the pumps’ inbuilt priming functionality.

A limitation of this study is that occlusions occurring at the start of an infusion were not taken into account. As our SD algorithm compares real-time pressure measurements to a predetermined set of baseline pressures and SD’s, it is equipped to handle situations where stable pressures are followed by an immediate pressure increase. Nevertheless the performance of our algorithms at the start of infusion remains to be tested. Likewise the possible advantages of defining baseline pressure values in real-time should be explored.

A future challenge is how to deal with pressure fluctuations resulting from factors other than occlusions. Pressures may fluctuate at the start of infusion, when the pump is moved (e.g. during transport) or when another pump connected to the same lumen is started. Clinical pump pressure data will be required to assess the robustness of our algorithms when such fluctuations occur.

In this study we used thin, rigid tubing that is commonly used in syringe pumps as well as thick, flexible tubing that is commonly used in volumetric pumps. When longer tubing is used a larger detection delay can be expected. We would expect that under such conditions of higher compliance our algorithms would also considerably reduce the time to occlusion detection. Additional studies are required to assess the performance of our algorithms under clinical conditions where many different tubing lengths may be used, as well as the possible impact of fluid viscosity and pressure fluctuations that may be due to external factors.

## Conclusions

Both the regression and SD algorithms were able to considerably reduce alarm delays in single-pump occlusion scenarios. The performance of the SD algorithm was superior to the regression algorithm in terms of alarm delay and robustness. During multi-pump occlusions the correlation algorithm reliably and very rapidly detected co-occlusions, which may also be useful to pin-point the segment of tubing in which an occlusion is present.

## Key concepts, definitions

Administration lumen: A hollow tube that allows for the delivery of a solution into the bloodstream of a patient. A CVC or PICC may have multiple lumens.

Central venous catheter (CVC): IV catheter consisting of one or more administration lumens, positioned in a central vein, allowing the continuous administration of concentrated or otherwise potentially damaging solutions.

Intravenous fluid: A fluid that is administered intravenously.

Intravenous (IV) therapy: The process of infusion of fluids into a vein of a patient.

Peripheral catheter: Single lumen intravenous catheter that is placed in a peripheral vein, which allows for the administration of solutions into the bloodstream of a patient.

Peripheral vein: Any vein not belonging to the major veins of the thorax or abdomen.

PICC: Peripherally inserted central catheter: a long intravenous catheter inserted in a peripheral vein but with the tip positioned in a large central vein

Solution: Intravenous fluid that may contain one or more drugs.

Syringe pump: A mechanical device used for the administration of infusion fluid to a patient by gradually displacing the plunger of a syringe by direct mechanical force. Typically delivers flows between 0.1 and 100 ml/h.

3L-CVC: A central venous catheter containing three lumens, therefore allowing for three separate flows of solutions into a central vein.

Volumetric pump: Infusion pump designed to deliver moderate to large infusion flows (i.e. 5 to 999 ml/hour).

## Supplementary Information

**Additional file 1.** Relationship between the window size (sec) and detection accuracy (%) for the SD algorithm.

**Additional file 2.** Calculated alarm delays in minutes for combinations of administration rates and pressure limits assuming a clinical baseline counter pressure of 150 mmHg.

## Data Availability

The datasets used and/or analyzed during the current study are available from the corresponding author on reasonable request.

## Abbreviations

CVC: Central venous catheter
ICU: Intensive care unit
IV: Intravenous
P(t): Pressure as a function of time
PVC: Peripheral venous catheter
SD: Standard deviation
Q(t): Flow rate as a function of time
