# Supplementary material for: Multi-infusion with integrated multiple pressure sensing allows earlier detection of line occlusions
Source: BMC Med Inform Decis Mak. 2021 Oct 28;21:295. doi: 10.1186/s12911-021-01668-7 (PMC8555317; doi:10.1186/s12911-021-01668-7)

Additional file to:

Multi-infusion with integrated multiple pressure sensing allows earlier detection of line occlusions.

Frank Doesburg[a*], Roy Oelen[a], Maurits H. Renes[a], Pedro M. Lourenço[b], Daan J. Touw[c,d], Maarten W. Nijsten[a].

[a]University of Groningen, University Medical Center Groningen, Department of Critical Care, Groningen, the Netherlands. Address: Hanzeplein 1, 9713 GZ Groningen, the Netherlands.

[b]Hanze Institute of Engineering, Assen, the Netherlands. Address: Industrieweg 1, 9402 NP, Assen, the Netherlands.

[c]University of Groningen, University Medical Center Groningen, Department of Clinical Pharmacy and Pharmacology, Groningen, the Netherlands. Address: Hanzeplein 1, 9713 GZ Groningen, the Netherlands.

[d]University of Groningen, Groningen Research Institute of Pharmacy, Department of Pharmaceutical Analysis, Groningen, the Netherlands. Address: Hanzeplein 1, 9713 GZ Groningen, the Netherlands.

*Corresponding author: Frank Doesburg

Postal address: UMCG Dpt. of Critical Care. Huispostcode TA29. Hanzeplein 1, 9713 GZ Groningen, The Netherlands.

E-mail: f.doesburg@umcg.nl.

Telephone: +31 (0) 50-3615650

Additional file 1. Relationship between the window size (sec) and detection accuracy (%) for the SD algorithm.

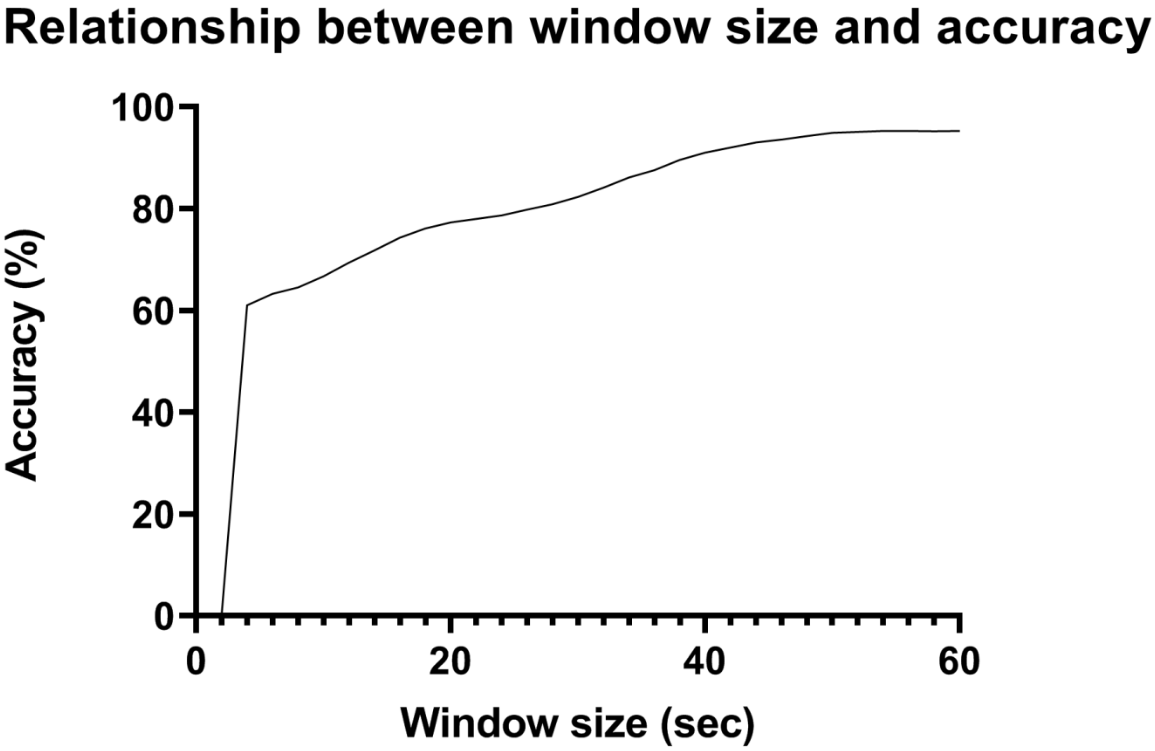

Supplement: Supplementary file 1 — Additional file 1. Relationship between the window size (sec) and detection accuracy (%) for the SD algorithm. [file 12911_2021_1668_MOESM1_ESM.pdf]
