# Supplementary material for: Multi-infusion with integrated multiple pressure sensing allows earlier detection of line occlusions
Source: BMC Med Inform Decis Mak. 2021 Oct 28;21:295. doi: 10.1186/s12911-021-01668-7 (PMC8555317; doi:10.1186/s12911-021-01668-7)
Supplement: Supplementary file 2 — Additional file 2. Calculated alarm delays in minutes for combinations of administration rates and pressure limits assuming a clinical baseline counter pressure of 150 mmHg. [file 12911_2021_1668_MOESM2_ESM.pdf]

Additional file to:

Multi-infusion with integrated multiple pressure sensing allows earlier detection of line occlusions.

Frank Doesburg[a*], Roy Oelen[a], Maurits H. Renes[a], Pedro M. Lourenço[b], Daan J. Touw[c,d], Maarten W. Nijsten[a].

[a]University of Groningen, University Medical Center Groningen, Department of Critical Care, Groningen, the Netherlands. Address: Hanzeplein 1, 9713 GZ Groningen, the Netherlands.

[b]Hanze Institute of Engineering, Assen, the Netherlands. Address: Industrieweg 1, 9402 NP, Assen, the Netherlands.

[c]University of Groningen, University Medical Center Groningen, Department of Clinical Pharmacy and Pharmacology, Groningen, the Netherlands. Address: Hanzeplein 1, 9713 GZ Groningen, the Netherlands.

[d]University of Groningen, Groningen Research Institute of Pharmacy, Department of Pharmaceutical Analysis, Groningen, the Netherlands. Address: Hanzeplein 1, 9713 GZ Groningen, the Netherlands.

*Corresponding author: Frank Doesburg

Postal address: UMCG Dpt. of Critical Care. Huispostcode TA29. Hanzeplein 1, 9713 GZ Groningen, The Netherlands.

E-mail: f.doesburg@umcg.nl.

Telephone: +31 (0) 50-3615650

Additional file 2. Calculated alarm delays in minutes for combinations of administration rates and pressure limits assuming a clinical baseline counter pressure of 150 mmHg.

	**Occlusion alarm threshold setting (mmHg)**					
	**300**	**400**	**500**	**600**	**700**	**800**
**Rate (ml/h)**	**Alarm delay mean ±SD (minutes)[1]**					
**1**	13.2 ±1.2	21.9 ±1.9	30.7 ±2.7	39.5 ±3.5	48.2 ±4.2	57.0 ±5.0
**2**	6.5 ±0.7	10.8 ±1.1	15.1 ±1.6	19.5 ±2.1	23.8 ±2.5	28.1 ±3.0
**4**	3.6 ±0.8	6.0 ±1.1	8.4 ±1.9	10.8 ±2.4	13.2 ±2.9	15.6 ±3.5
**8**	1.6 ±0.1	2.7 ±0.2	3.8 ±0.2	4.9 ±0.3	6.0 ±0.4	7.1 ±0.4
**16**	0.9 ±0.1	1.5 ±0.1	2.1 ±0.2	2.7 ±0.2	3.2 ±0.3	3.8 ±0.3
**32**	0.8 ±0.3	1.4 ±0.5	2.0 ±0.7	2.5 ±0.9	3.1 ±1.2	3.7 ±1.4
**[1]Calculated using linear regression assuming a clinical baseline pressure of 150 mmHg**						
